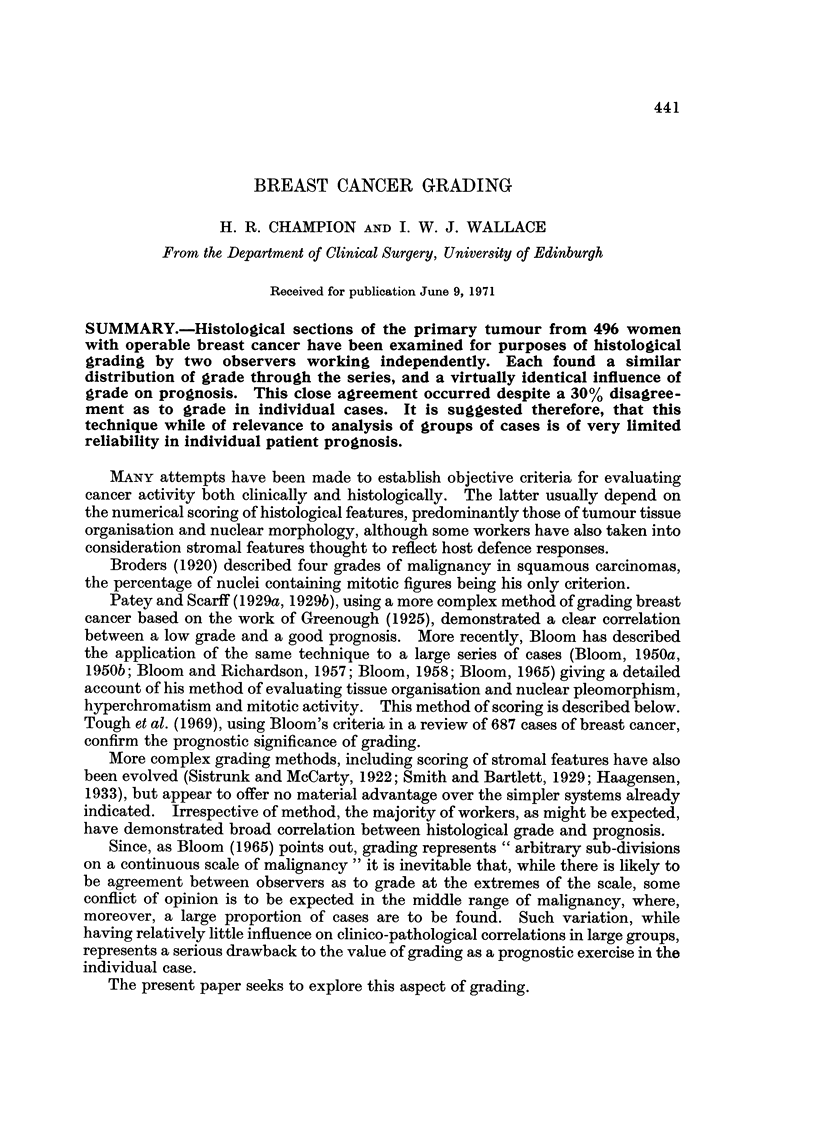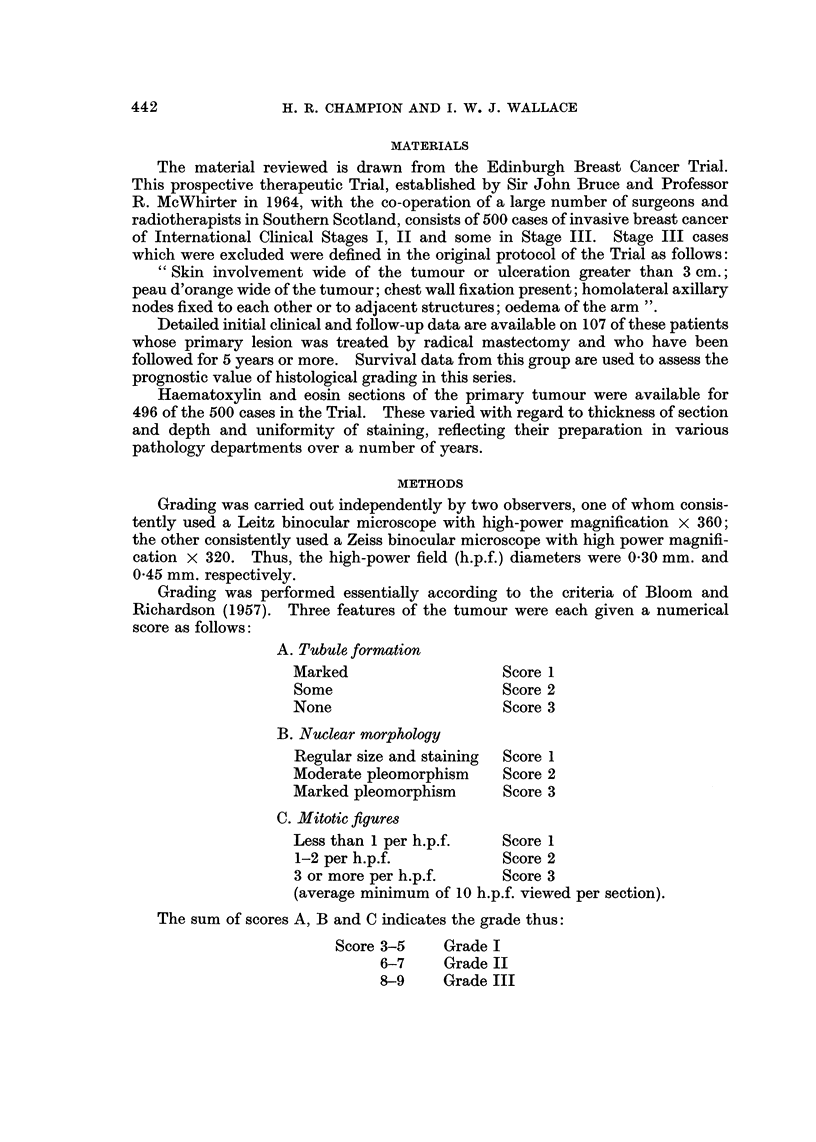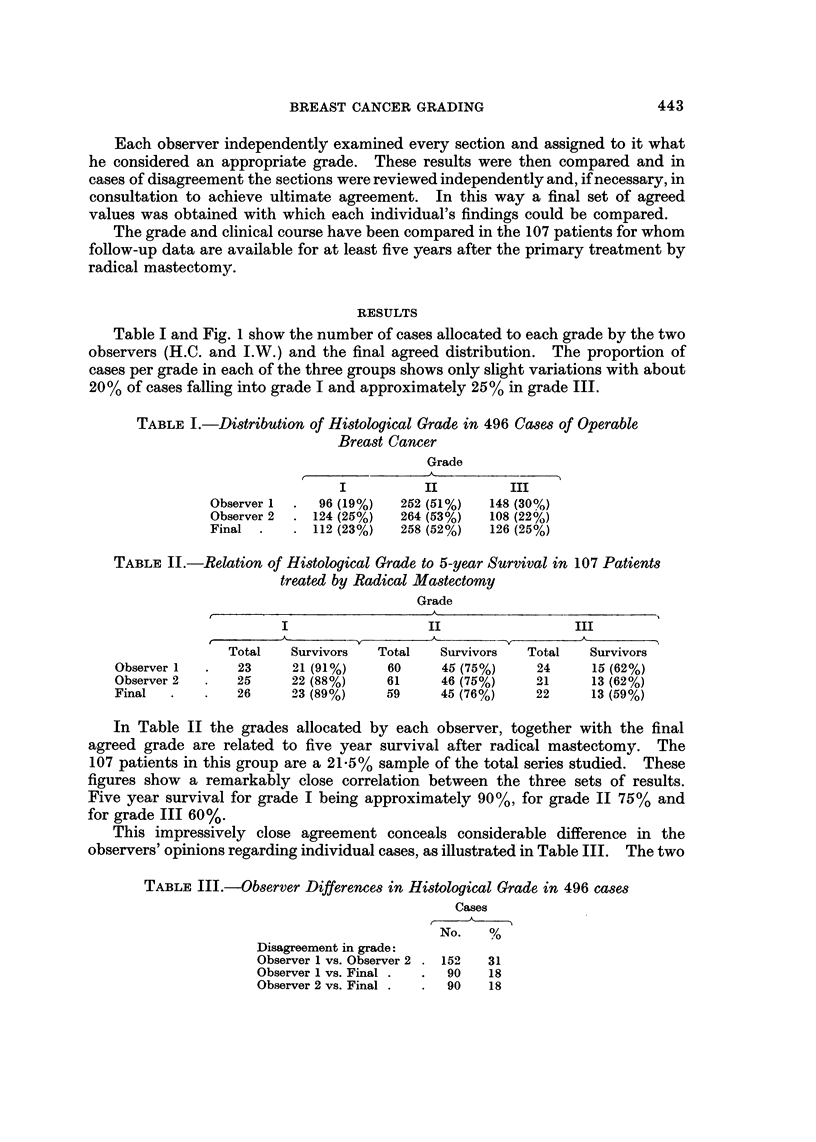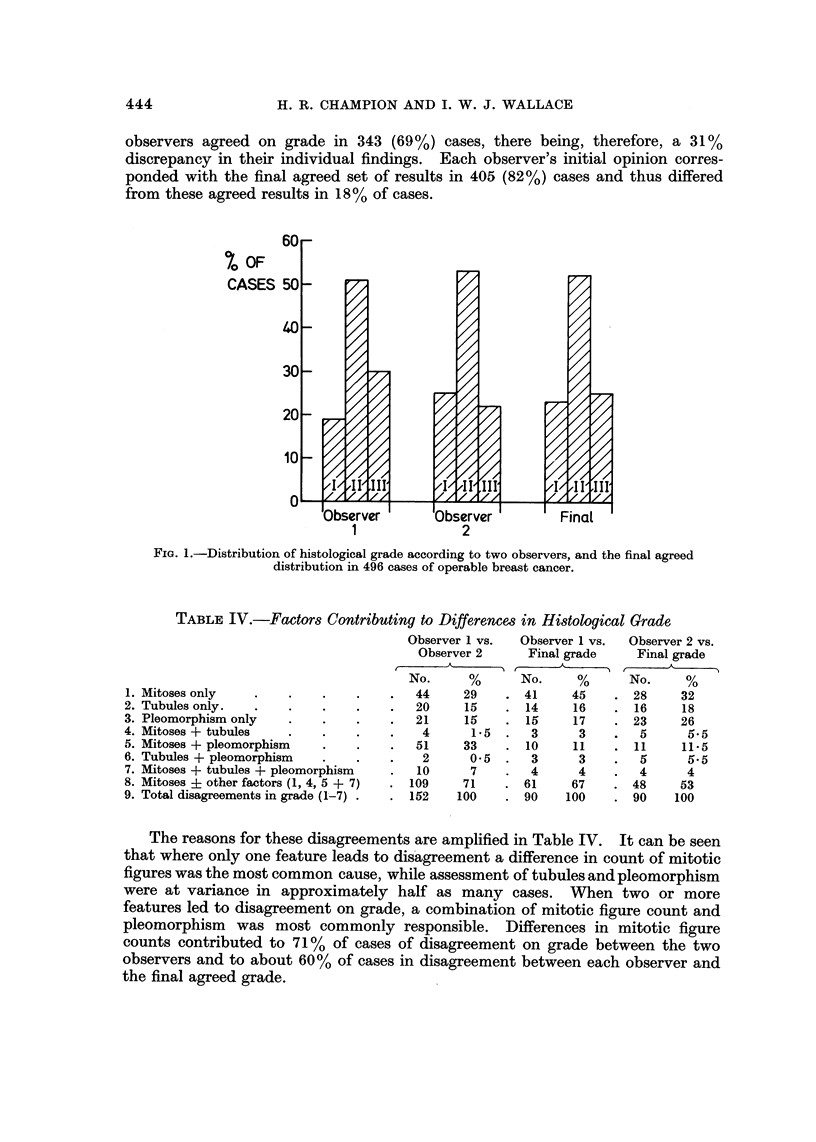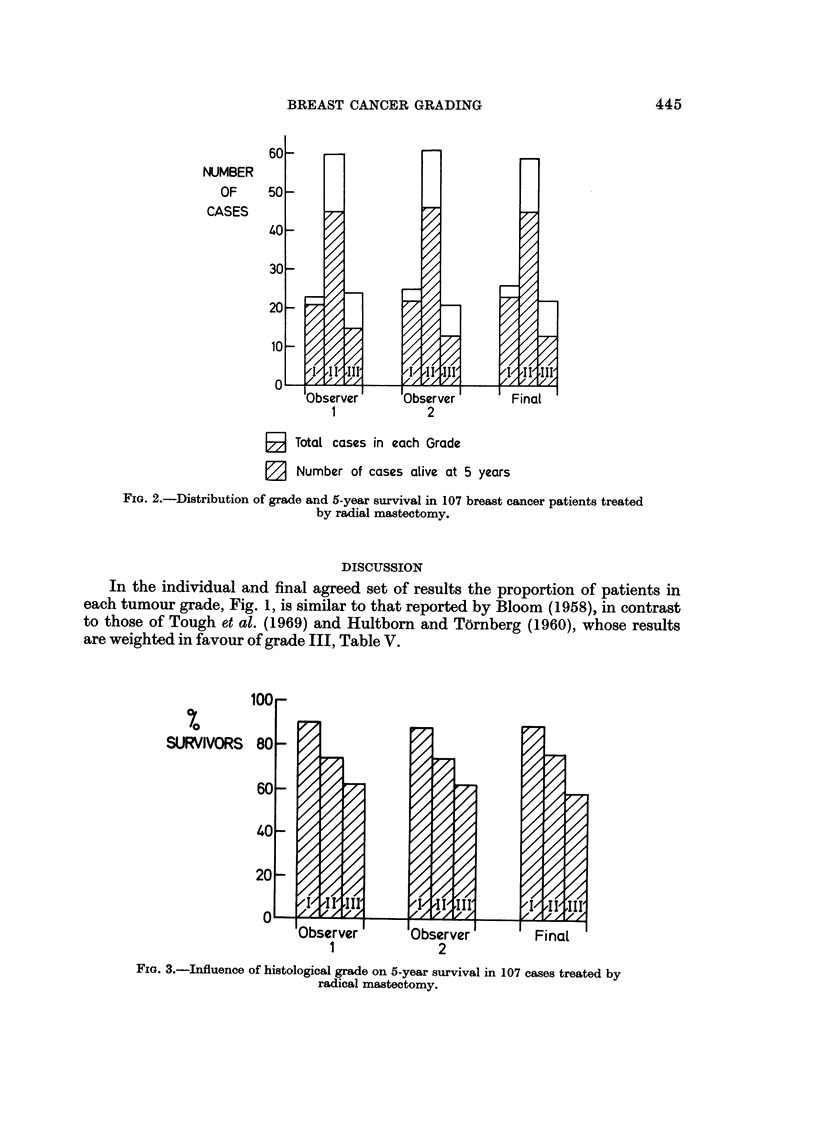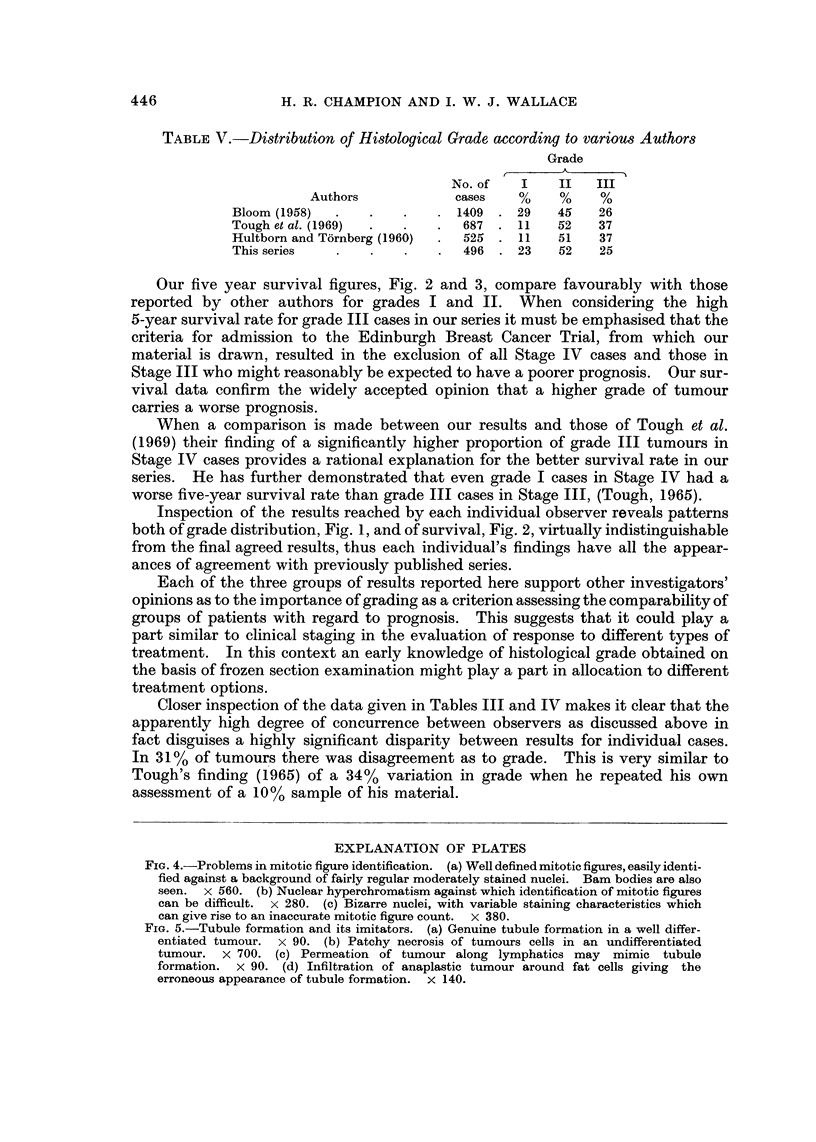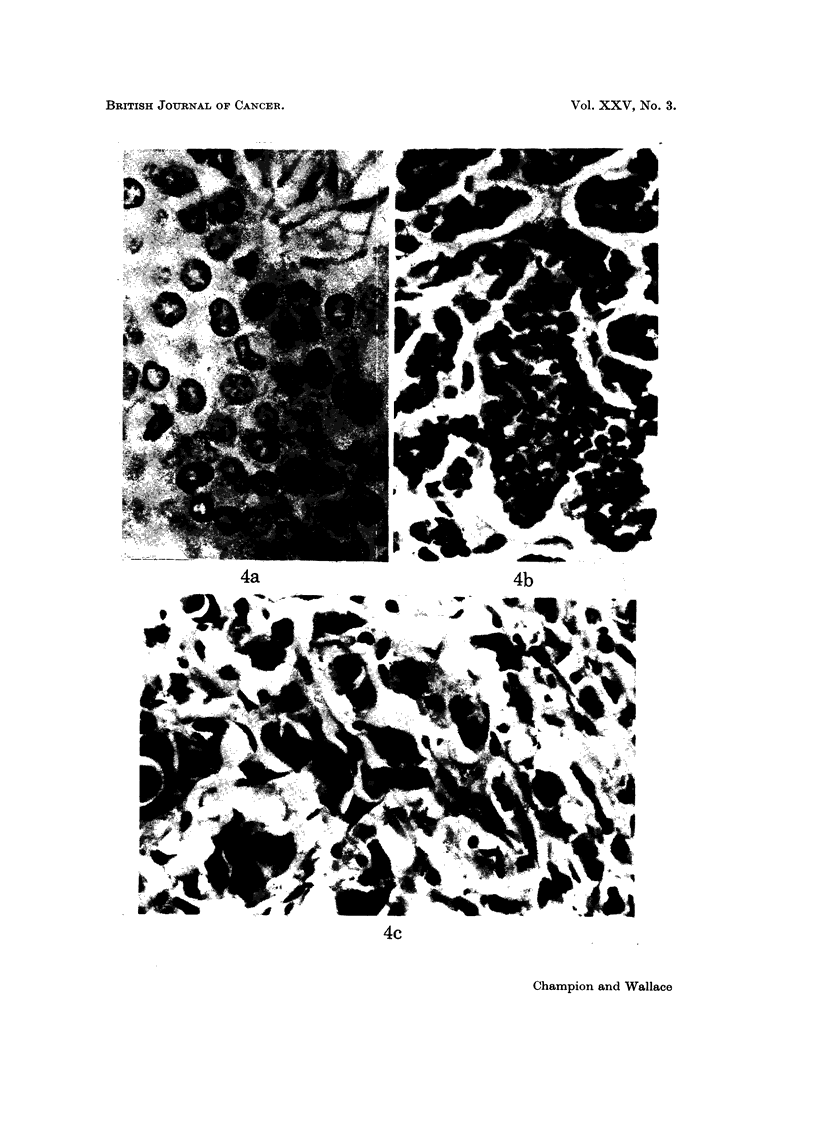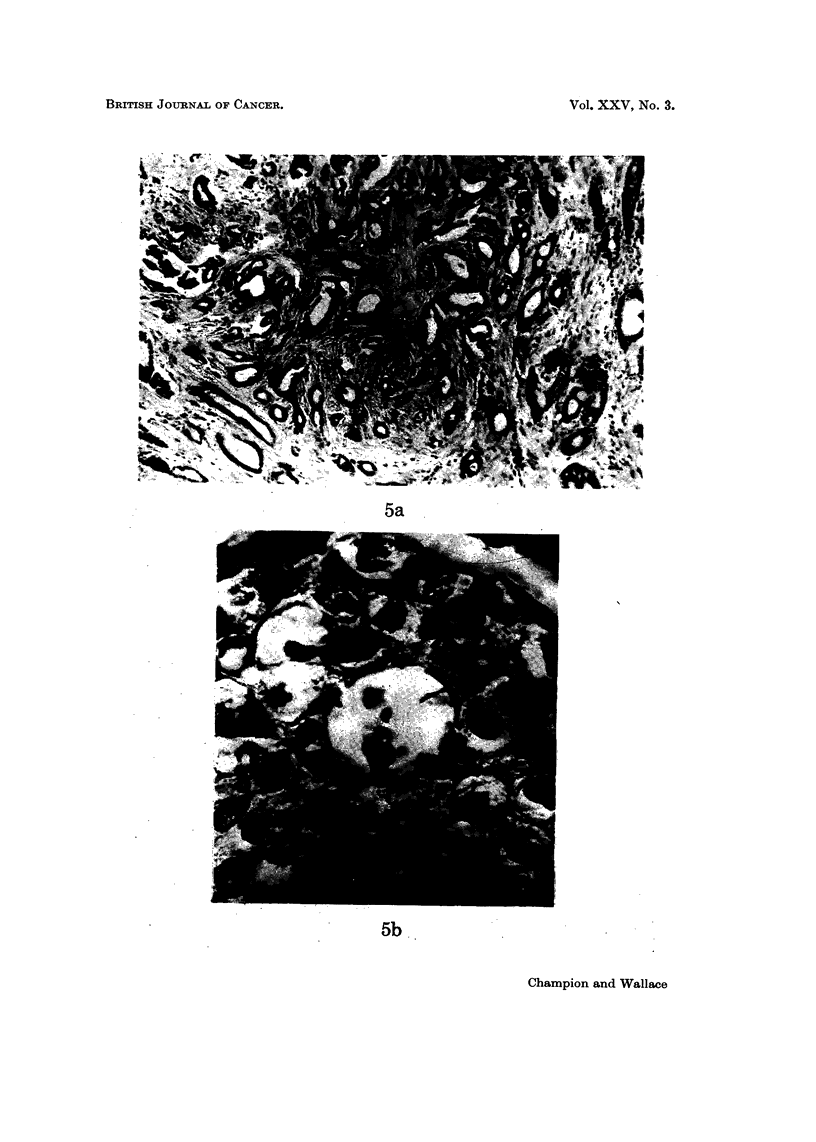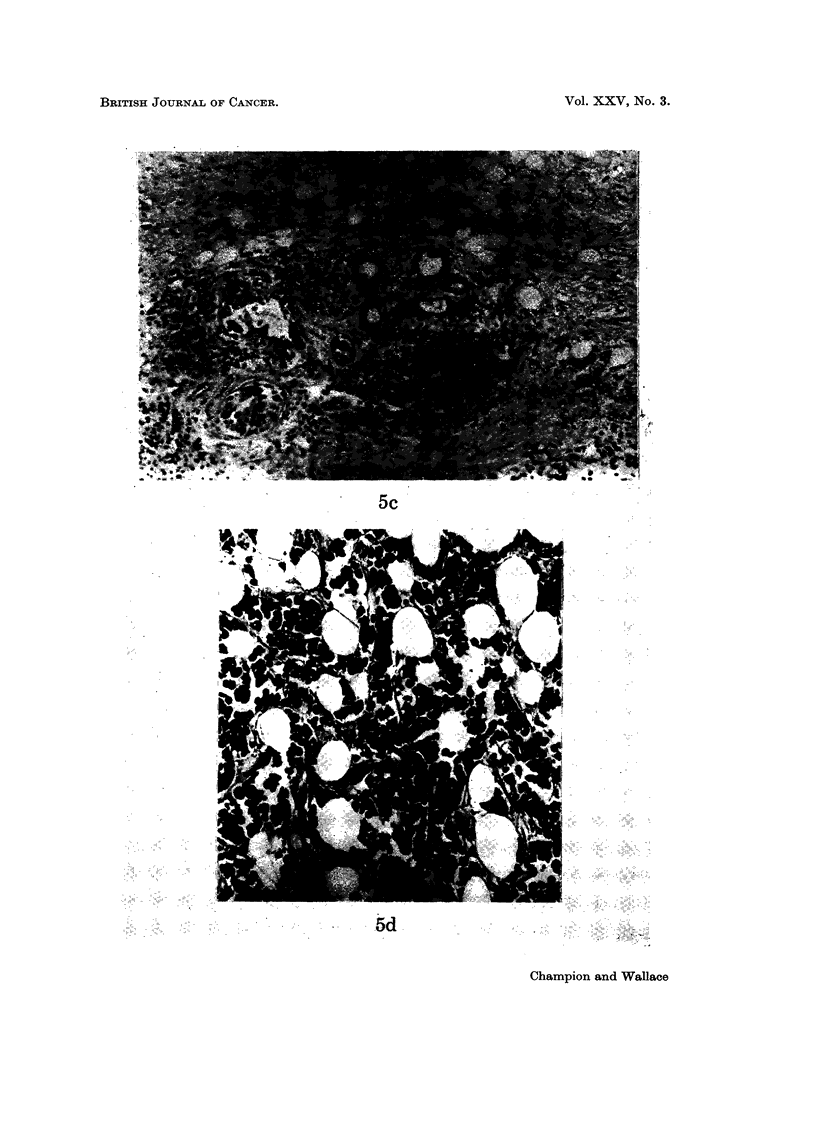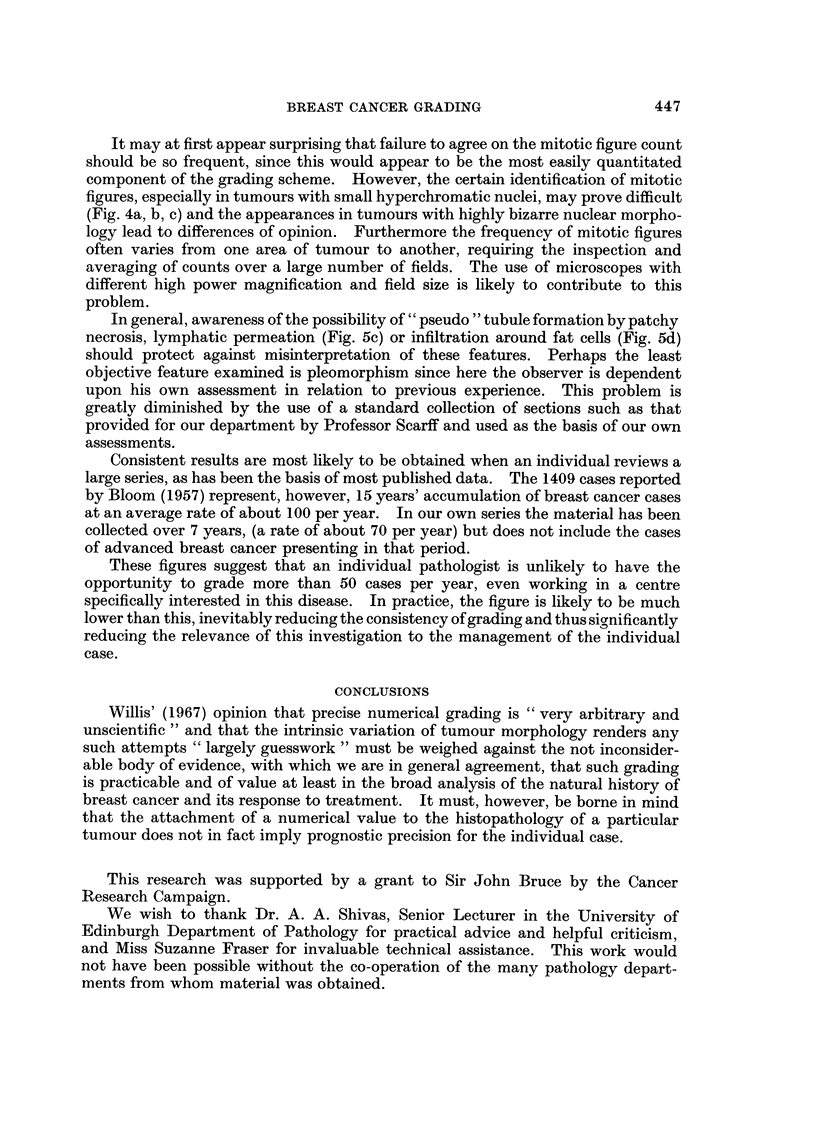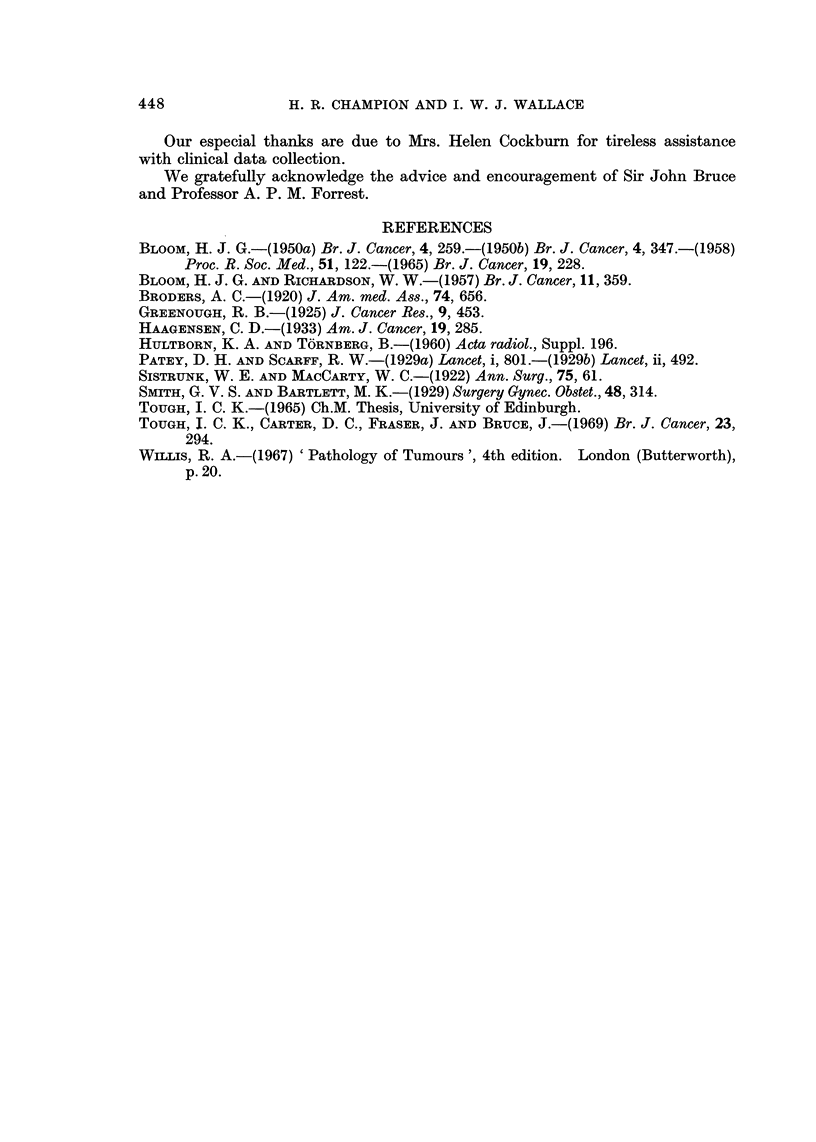# Breast Cancer Grading

**DOI:** 10.1038/bjc.1971.56

**Published:** 1971-09

**Authors:** H. R. Champion, I. W. J. Wallace

## Abstract

**Images:**


### BREAST CANCER GRADING

H. R. CHAMPION AND I. W. J. WALLACE

*From the Department of Clinical Surgery, University of Edinburgh*

Received for publication June 9, 1971

**SUMMARY.—Histological sections of the primary tumour from 496 women with operable breast cancer have been examined for purposes of histological grading by two observers working independently. Each found a similar distribution of grade through the series, and a virtually identical influence of grade on prognosis. This close agreement occurred despite a 30% disagreement as to grade in individual cases. It is suggested therefore, that this technique while of relevance to analysis of groups of cases is of very limited reliability in individual patient prognosis.**

MANY attempts have been made to establish objective criteria for evaluating cancer activity both clinically and histologically. The latter usually depend on the numerical scoring of histological features, predominantly those of tumour tissue organisation and nuclear morphology, although some workers have also taken into consideration stromal features thought to reflect host defence responses.

Broders (1920) described four grades of malignancy in squamous carcinomas, the percentage of nuclei containing mitotic figures being his only criterion.

Patey and Scarff (1929*a*, 1929*b*), using a more complex method of grading breast cancer based on the work of Greenough (1925), demonstrated a clear correlation between a low grade and a good prognosis. More recently, Bloom has described the application of the same technique to a large series of cases (Bloom, 1950*a*, 1950*b*; Bloom and Richardson, 1957; Bloom, 1958; Bloom, 1965) giving a detailed account of his method of evaluating tissue organisation and nuclear pleomorphism, hyperchromatism and mitotic activity. This method of scoring is described below. Tough *et al.* (1969), using Bloom's criteria in a review of 687 cases of breast cancer, confirm the prognostic significance of grading.

More complex grading methods, including scoring of stromal features have also been evolved (Sistrunk and McCarty, 1922; Smith and Bartlett, 1929; Haagensen, 1933), but appear to offer no material advantage over the simpler systems already indicated. Irrespective of method, the majority of workers, as might be expected, have demonstrated broad correlation between histological grade and prognosis.

Since, as Bloom (1965) points out, grading represents " arbitrary sub-divisions on a continuous scale of malignancy " it is inevitable that, while there is likely to be agreement between observers as to grade at the extremes of the scale, some conflict of opinion is to be expected in the middle range of malignancy, where, moreover, a large proportion of cases are to be found. Such variation, while having relatively little influence on clinico-pathological correlations in large groups, represents a serious drawback to the value of grading as a prognostic exercise in the individual case.

The present paper seeks to explore this aspect of grading.

### MATERIALS

The material reviewed is drawn from the Edinburgh Breast Cancer Trial. This prospective therapeutic Trial, established by Sir John Bruce and Professor R. McWhirter in 1964, with the co-operation of a large number of surgeons and radiotherapists in Southern Scotland, consists of 500 cases of invasive breast cancer of International Clinical Stages I, II and some in Stage III. Stage III cases which were excluded were defined in the original protocol of the Trial as follows:

" Skin involvement wide of the tumour or ulceration greater than 3 cm.; peau d'orange wide of the tumour; chest wall fixation present; homolateral axillary nodes fixed to each other or to adjacent structures; oedema of the arm ".

Detailed initial clinical and follow-up data are available on 107 of these patients whose primary lesion was treated by radical mastectomy and who have been followed for 5 years or more. Survival data from this group are used to assess the prognostic value of histological grading in this series.

Haematoxylin and eosin sections of the primary tumour were available for 496 of the 500 cases in the Trial. These varied with regard to thickness of section and depth and uniformity of staining, reflecting their preparation in various pathology departments over a number of years.

### METHODS

Grading was carried out independently by two observers, one of whom consistently used a Leitz binocular microscope with high-power magnification × 360; the other consistently used a Zeiss binocular microscope with high power magnification × 320. Thus, the high-power field (h.p.f.) diameters were 0·30 mm. and 0·45 mm. respectively.

Grading was performed essentially according to the criteria of Bloom and Richardson (1957). Three features of the tumour were each given a numerical score as follows:

A. *Tubule formation*

| | |
|---|---|
| Marked | Score 1 |
| Some | Score 2 |
| None | Score 3 |

B. *Nuclear morphology*

| | |
|---|---|
| Regular size and staining | Score 1 |
| Moderate pleomorphism | Score 2 |
| Marked pleomorphism | Score 3 |

C. *Mitotic figures*

| | |
|---|---|
| Less than 1 per h.p.f. | Score 1 |
| 1–2 per h.p.f. | Score 2 |
| 3 or more per h.p.f. | Score 3 |

(average minimum of 10 h.p.f. viewed per section).

The sum of scores A, B and C indicates the grade thus:

| | |
|---|---|
| Score 3–5 | Grade I |
| 6–7 | Grade II |
| 8–9 | Grade III |

Each observer independently examined every section and assigned to it what he considered an appropriate grade. These results were then compared and in cases of disagreement the sections were reviewed independently and, if necessary, in consultation to achieve ultimate agreement. In this way a final set of agreed values was obtained with which each individual's findings could be compared.

The grade and clinical course have been compared in the 107 patients for whom follow-up data are available for at least five years after the primary treatment by radical mastectomy.

### RESULTS

Table I and Fig. 1 show the number of cases allocated to each grade by the two observers (H.C. and I.W.) and the final agreed distribution. The proportion of cases per grade in each of the three groups shows only slight variations with about 20% of cases falling into grade I and approximately 25% in grade III.

TABLE I.—*Distribution of Histological Grade in* 496 *Cases of Operable Breast Cancer*

| | Grade | | |
|---|---|---|---|
| | I | II | III |
| Observer 1 | 96 (19%) | 252 (51%) | 148 (30%) |
| Observer 2 | 124 (25%) | 264 (53%) | 108 (22%) |
| Final | 112 (23%) | 258 (52%) | 126 (25%) |

TABLE II.—*Relation of Histological Grade to 5-year Survival in* 107 *Patients treated by Radical Mastectomy*

| | Grade | | | | | |
|---|---|---|---|---|---|---|
| | I | | II | | III | |
| | Total | Survivors | Total | Survivors | Total | Survivors |
| Observer 1 | 23 | 21 (91%) | 60 | 45 (75%) | 24 | 15 (62%) |
| Observer 2 | 25 | 22 (88%) | 61 | 46 (75%) | 21 | 13 (62%) |
| Final | 26 | 23 (89%) | 59 | 45 (76%) | 22 | 13 (59%) |

In Table II the grades allocated by each observer, together with the final agreed grade are related to five year survival after radical mastectomy. The 107 patients in this group are a 21·5% sample of the total series studied. These figures show a remarkably close correlation between the three sets of results. Five year survival for grade I being approximately 90%, for grade II 75% and for grade III 60%.

This impressively close agreement conceals considerable difference in the observers' opinions regarding individual cases, as illustrated in Table III. The two

TABLE III.—*Observer Differences in Histological Grade in* 496 *cases*

| | Cases | |
|---|---|---|
| | No. | % |
| Disagreement in grade: | | |
| Observer 1 vs. Observer 2 | 152 | 31 |
| Observer 1 vs. Final | 90 | 18 |
| Observer 2 vs. Final | 90 | 18 |

observers agreed on grade in 343 (69%) cases, there being, therefore, a 31% discrepancy in their individual findings. Each observer's initial opinion corresponded with the final agreed set of results in 405 (82%) cases and thus differed from these agreed results in 18% of cases.

FIG. 1.—Distribution of histological grade according to two observers, and the final agreed distribution in 496 cases of operable breast cancer.

TABLE IV.—*Factors Contributing to Differences in Histological Grade*

| | Observer 1 vs. Observer 2 | | Observer 1 vs. Final grade | | Observer 2 vs. Final grade | |
|---|---|---|---|---|---|---|
| | No. | % | No. | % | No. | % |
| 1. Mitoses only | 44 | 29 | 41 | 45 | 28 | 32 |
| 2. Tubules only | 20 | 15 | 14 | 16 | 16 | 18 |
| 3. Pleomorphism only | 21 | 15 | 15 | 17 | 23 | 26 |
| 4. Mitoses + tubules | 4 | 1·5 | 3 | 3 | 5 | 5·5 |
| 5. Mitoses + pleomorphism | 51 | 33 | 10 | 11 | 11 | 11·5 |
| 6. Tubules + pleomorphism | 2 | 0·5 | 3 | 3 | 5 | 5·5 |
| 7. Mitoses + tubules + pleomorphism | 10 | 7 | 4 | 4 | 4 | 4 |
| 8. Mitoses ± other factors (1, 4, 5 + 7) | 109 | 71 | 61 | 67 | 48 | 53 |
| 9. Total disagreements in grade (1–7) | 152 | 100 | 90 | 100 | 90 | 100 |

The reasons for these disagreements are amplified in Table IV. It can be seen that where only one feature leads to disagreement a difference in count of mitotic figures was the most common cause, while assessment of tubules and pleomorphism were at variance in approximately half as many cases. When two or more features led to disagreement on grade, a combination of mitotic figure count and pleomorphism was most commonly responsible. Differences in mitotic figure counts contributed to 71% of cases of disagreement on grade between the two observers and to about 60% of cases in disagreement between each observer and the final agreed grade.

FIG. 2.—Distribution of grade and 5-year survival in 107 breast cancer patients treated by radial mastectomy.

### DISCUSSION

In the individual and final agreed set of results the proportion of patients in each tumour grade, Fig. 1, is similar to that reported by Bloom (1958), in contrast to those of Tough *et al.* (1969) and Hultborn and Törnberg (1960), whose results are weighted in favour of grade III, Table V.

FIG. 3.—Influence of histological grade on 5-year survival in 107 cases treated by radical mastectomy.

TABLE V.—*Distribution of Histological Grade according to various Authors*

| Authors | No. of cases | Grade I % | Grade II % | Grade III % |
|---|---|---|---|---|
| Bloom (1958) | 1409 | 29 | 45 | 26 |
| Tough *et al.* (1969) | 687 | 11 | 52 | 37 |
| Hultborn and Törnberg (1960) | 525 | 11 | 51 | 37 |
| This series | 496 | 23 | 52 | 25 |

Our five year survival figures, Fig. 2 and 3, compare favourably with those reported by other authors for grades I and II. When considering the high 5-year survival rate for grade III cases in our series it must be emphasised that the criteria for admission to the Edinburgh Breast Cancer Trial, from which our material is drawn, resulted in the exclusion of all Stage IV cases and those in Stage III who might reasonably be expected to have a poorer prognosis. Our survival data confirm the widely accepted opinion that a higher grade of tumour carries a worse prognosis.

When a comparison is made between our results and those of Tough *et al.* (1969) their finding of a significantly higher proportion of grade III tumours in Stage IV cases provides a rational explanation for the better survival rate in our series. He has further demonstrated that even grade I cases in Stage IV had a worse five-year survival rate than grade III cases in Stage III, (Tough, 1965).

Inspection of the results reached by each individual observer reveals patterns both of grade distribution, Fig. 1, and of survival, Fig. 2, virtually indistinguishable from the final agreed results, thus each individual's findings have all the appearances of agreement with previously published series.

Each of the three groups of results reported here support other investigators' opinions as to the importance of grading as a criterion assessing the comparability of groups of patients with regard to prognosis. This suggests that it could play a part similar to clinical staging in the evaluation of response to different types of treatment. In this context an early knowledge of histological grade obtained on the basis of frozen section examination might play a part in allocation to different treatment options.

Closer inspection of the data given in Tables III and IV makes it clear that the apparently high degree of concurrence between observers as discussed above in fact disguises a highly significant disparity between results for individual cases. In 31% of tumours there was disagreement as to grade. This is very similar to Tough's finding (1965) of a 34% variation in grade when he repeated his own assessment of a 10% sample of his material.

---

### EXPLANATION OF PLATES

FIG. 4.—Problems in mitotic figure identification. (a) Well defined mitotic figures, easily identified against a background of fairly regular moderately stained nuclei. Bam bodies are also seen. × 560. (b) Nuclear hyperchromatism against which identification of mitotic figures can be difficult. × 280. (c) Bizarre nuclei, with variable staining characteristics which can give rise to an inaccurate mitotic figure count. × 380.

FIG. 5.—Tubule formation and its imitators. (a) Genuine tubule formation in a well differentiated tumour. × 90. (b) Patchy necrosis of tumours cells in an undifferentiated tumour. × 700. (c) Permeation of tumour along lymphatics may mimic tubule formation. × 90. (d) Infiltration of anaplastic tumour around fat cells giving the erroneous appearance of tubule formation. × 140.

4a 4b

4c

Champion and Wallace

5a

5b

Champion and Wallace

5c

5d

Champion and Wallace

It may at first appear surprising that failure to agree on the mitotic figure count should be so frequent, since this would appear to be the most easily quantitated component of the grading scheme. However, the certain identification of mitotic figures, especially in tumours with small hyperchromatic nuclei, may prove difficult (Fig. 4a, b, c) and the appearances in tumours with highly bizarre nuclear morphology lead to differences of opinion. Furthermore the frequency of mitotic figures often varies from one area of tumour to another, requiring the inspection and averaging of counts over a large number of fields. The use of microscopes with different high power magnification and field size is likely to contribute to this problem.

In general, awareness of the possibility of " pseudo " tubule formation by patchy necrosis, lymphatic permeation (Fig. 5c) or infiltration around fat cells (Fig. 5d) should protect against misinterpretation of these features. Perhaps the least objective feature examined is pleomorphism since here the observer is dependent upon his own assessment in relation to previous experience. This problem is greatly diminished by the use of a standard collection of sections such as that provided for our department by Professor Scarff and used as the basis of our own assessments.

Consistent results are most likely to be obtained when an individual reviews a large series, as has been the basis of most published data. The 1409 cases reported by Bloom (1957) represent, however, 15 years' accumulation of breast cancer cases at an average rate of about 100 per year. In our own series the material has been collected over 7 years, (a rate of about 70 per year) but does not include the cases of advanced breast cancer presenting in that period.

These figures suggest that an individual pathologist is unlikely to have the opportunity to grade more than 50 cases per year, even working in a centre specifically interested in this disease. In practice, the figure is likely to be much lower than this, inevitably reducing the consistency of grading and thus significantly reducing the relevance of this investigation to the management of the individual case.

### CONCLUSIONS

Willis' (1967) opinion that precise numerical grading is " very arbitrary and unscientific " and that the intrinsic variation of tumour morphology renders any such attempts " largely guesswork " must be weighed against the not inconsiderable body of evidence, with which we are in general agreement, that such grading is practicable and of value at least in the broad analysis of the natural history of breast cancer and its response to treatment. It must, however, be borne in mind that the attachment of a numerical value to the histopathology of a particular tumour does not in fact imply prognostic precision for the individual case.

This research was supported by a grant to Sir John Bruce by the Cancer Research Campaign.

We wish to thank Dr. A. A. Shivas, Senior Lecturer in the University of Edinburgh Department of Pathology for practical advice and helpful criticism, and Miss Suzanne Fraser for invaluable technical assistance. This work would not have been possible without the co-operation of the many pathology departments from whom material was obtained.

Our especial thanks are due to Mrs. Helen Cockburn for tireless assistance with clinical data collection.

We gratefully acknowledge the advice and encouragement of Sir John Bruce and Professor A. P. M. Forrest.

### REFERENCES

Bloom, H. J. G.—(1950*a*) *Br. J. Cancer*, **4**, 259.—(1950*b*) *Br. J. Cancer*, **4**, 347.—(1958) *Proc. R. Soc. Med.*, **51**, 122.—(1965) *Br. J. Cancer*, **19**, 228.

Bloom, H. J. G. and Richardson, W. W.—(1957) *Br. J. Cancer*, **11**, 359.

Broders, A. C.—(1920) *J. Am. med. Ass.*, **74**, 656.

Greenough, R. B.—(1925) *J. Cancer Res.*, **9**, 453.

Haagensen, C. D.—(1933) *Am. J. Cancer*, **19**, 285.

Hultborn, K. A. and Törnberg, B.—(1960) *Acta radiol.*, Suppl. 196.

Patey, D. H. and Scarff, R. W.—(1929*a*) *Lancet*, i, 801.—(1929*b*) *Lancet*, ii, 492.

Sistrunk, W. E. and MacCarty, W. C.—(1922) *Ann. Surg.*, **75**, 61.

Smith, G. V. S. and Bartlett, M. K.—(1929) *Surgery Gynec. Obstet.*, **48**, 314.

Tough, I. C. K.—(1965) Ch.M. Thesis, University of Edinburgh.

Tough, I. C. K., Carter, D. C., Fraser, J. and Bruce, J.—(1969) *Br. J. Cancer*, **23**, 294.

Willis, R. A.—(1967) ' Pathology of Tumours ', 4th edition. London (Butterworth), p. 20.